# Development and Application of Novel SSR Markers to Assess the Genetic Diversity and Population Structure of *Phacelia secunda* Along an Altitudinal Gradient in the Central Chile Andes

**DOI:** 10.3390/plants14071135

**Published:** 2025-04-05

**Authors:** Cristian Torres-Díaz, Ana Ortíz-Sepúlveda, Moisés A. Valladares, Darío Farias-Cantillana, Marco A. Molina-Montenegro, Gabriel I. Ballesteros

**Affiliations:** 1 Grupo de Investigación en Biodiversidad & Cambio Global (GIBCG), Departamento de Ciencias Básicas, Universidad del Bío-Bío, Chillan 3800708, Chile; 2 Laboratorio de Biología Evolutiva, Facultad de Ciencias Biológicas, Pontificia Universidad Católica de Chile, Santiago 7820436, Chile; 3 Instituto de Ciencias Biológicas, Universidad de Talca, Avenida Lircay s/n, Talca 3460000, Chile; 4 Centro de Investigación en Estudios Avanzados del Maule (CIEAM), Universidad Católica del Maule, Talca 3460000, Chile

**Keywords:** high-elevation ecosystems, high-throughput sequencing, mountain ecosystems, alpine, Andean, SSRs

## Abstract

*Phacelia secunda* J.F. Gmel. (Boraginaceae) is a widely distributed insect-pollinated perennial herb. In central Chile (33° S), it occurs from the sea level up to 3600 m in the Andes, exhibiting broad morphological variation. In this study, we developed and characterized novel polymorphic microsatellites for this species, using an Illimina MiSeq sequencing platform. Nineteen polymorphic loci were obtained, with alleles numbers ranging from 3 to 13 per locus (mean = 5.84). Observed (*H*_O_) and expected heterozygosities (*H*_E_) ranged from 0.050 to 0.900 and from 0.049 to 0.825, respectively. These markers were applied to assess the genetic diversity and population structure along an altitudinal spanning from 1600 to 3600 m. The highest elevation population exhibited significantly lower within-population genetic diversity compared to lower-elevation populations. Significant population differentiation was observed along the gradient. Gene flow estimates support a stepping-stone like mode of migration, with greater exchange between adjacent elevations. These new microsatellites provide a valuable tool for elucidating the influence of altitude on genetic diversity and structure, and for evaluating the roles of local adaptation and phenotypic plasticity in shaping population variation.

## 1. Introduction

*Phacelia secunda* J.F. Gmel. (Boraginaceae) is an insect-pollinated perennial herb distributed in Argentina, Chile and Bolivia. In Chile, it occurs over a wide latitudinal range —from Parinacota (18° S) to Tierra del Fuego (54° S)—and across a broad altitudinal, from sea level up to the vegetation limit above the tree line. This broad altitudinal distribution is accompanied by marked morphological variation. For example, in the Andes of central Chile (33° S), ref. [1] documented that rosette diameter, the number of inflorescences per plant, and the longitude of pubescence increased with elevation, while the height of inflorescences decreased [1,2]. In addition, investigations of the photosynthetic responses of *P. secunda* plants from populations at 1600, 2800 and 3600 m, grown under common garden conditions, have revealed a low plasticity in response to future climate change [3].

Given its broad distribution and associated morphological variability, *P. secunda* represents an excellent model to test hypotheses related to its ability to colonize diverse environments and to evaluate the effects of elevation on its genetic diversity and population differentiation [4,5,6]. Furthermore, this species is well suited for addressing additional questions about the impacts of elevation on plant mating systems, such as the increased pollination probability hypothesis in high-elevation ecosystems [7,8], which has rarely been tested using molecular markers to estimate changes in outcrossing rates [9]. Similarly, this study system provides an opportunity for gaining insights on the impacts of phenological segregations [10,11] on genetic diversity, population differentiation and gene flow along elevational gradients. In addition, this species is also ideal to investigate the relative contributions of local adaptation and phenotypic plasticity, and their interactions, for maintaining viable populations across altitudinal and latitudinal gradients. However, the lack of species-specific molecular markers for *P. secunda* has hindered progress in these research areas.

Despite the increasing affordability of high-throughput sequencing (HTS) technologies, microsatellite markers (also known as simple sequence repeats, SSRs), remain valuable tools in molecular ecology and conservation biology, especially for non-model species [12]. SSRs are co-dominant, allowing for the distinction between homozygotes and heterozygotes, and are composed of tandem repeats of two to five nucleotides. Their high level of polymorphism, ease of use, reproducibility and cost-effectiveness make them particularly useful for applications such as estimating outcrossing rates, conducting parentage analyses, and assessing population structure and evolutionary processes [13,14]. Although single nucleotide polymorphisms (SNPs) have prominence in ecological, evolutionary, and conservation studies, SSRs remain a cost-effective and efficient option for many research objectives [12].

The tendency toward horizontal growth instead of vertical growth at higher elevations was interpreted as an adaptation to the cold and harsh environmental conditions characteristic of high-elevation environments [1,2]. Under the hypothesis of local adaptation, high-elevation populations are expected to be genetically isolated from lower-elevation populations, as gene flow would otherwise counteract the effects of natural selection and diminish the influence of locally adapted alleles on the overall fitness [15]. Alternatively, the absence of genetic differentiation along the gradient would suggest that the observed morphological changes are primarily driven by environmentally induced plastic responses, including phenotypic plasticity and epigenetic modifications rather than by genetic divergence [16,17,18,19]. Therefore, the development of molecular markers for *P. secunda* is essential to assess genetic differentiation and gene flow along altitudinal gradients and to explore the possibility of local adaptation.

The patterns of within-population genetic diversity along altitudinal gradients are highly variable and have been attributed to the interplay of demographic processes such as genetic drift, bottlenecks, and gene flow [4,20]. As stated by [20], all possible outcomes have been found: (i) greater within-population genetic diversity at intermediate altitudes, e.g., [5]; (ii) lower within-population genetic diversity at higher elevation, e.g., [21,22]; (iii) greater within-population genetic diversity at higher elevation, e.g., [23,24]; and (iv) no effect of altitude on within-population genetic diversity, e.g., [4,25]. Molecular markers, therefore, can provide critical insights into the evolutionary consequences of these patterns in *P. secunda*.

In the present study, we performed de novo DNA sequencing of a pool of individuals of *Phacelia secunda* using high-throughput Illumina sequencing technology. From the assembled genomic data, we identified polymorphic SSR loci, primer pairs, and characterized 19 novel SSR markers. The cross-species transferability of these markers was evaluated in the closely related annual species *Phacelia brachyantha*. Additionally, the newly developed SSR markers were employed to assess the genetic diversity and population structure of *P. secunda* along an altitudinal gradient in the Andes of central Chile (33° S), spanning elevations from 1600 to 3600 m.

## 2. Results

### 2.1. Microsatellite Screening

A total of 1,917,566 reads with an average length of 435 bases were obtained from the shotgun sequencing. From these reads, 2015 microsatellites were identified, of which 28 were initially considered putatively polymorphic. Primer pairs were designed and synthesized for all loci; however, only 19 were correctly amplified and polymorphic. While nine markers were di-nucleotide, the other ten were trinucleotide with sizes that ranged from 310 to 560 bp (Table S1).

### 2.2. Microsatellite Loci Characterization

Considering the entire dataset, a total of 92 alleles were scored. The total number of alleles per locus (*A*_T_) ranged from 3 to 10, with a mean of 5.421 (Table S2). Considering the entire dataset, the mean number of alleles per locus (*A*_E_) and the number of effective alleles per locus (*A*_E_) were 4.737 and 2.574, respectively (Table 1). A total of 23 private alleles were found, six for the lower elevation, nine for the mid-low elevation, seven for the mid-high elevation and one for the higher elevation. For the entire dataset, the observed (*H*_O_) and expected (*H*_E_) heterozygosity ranged from 0.000 to 0.933 and from 0.039 to 0.843, respectively, with a global average of 0.439 and 0.502 for *H*_O_ and *H*_E_, respectively (Table 1). The global inbreeding was *F* = 0.110, mostly due to greater heterozygous deficiency in populations low and high (Table 1).

Evidence of null alleles was found in nine, three, four and four loci in the low, mid-low, mid-high and high populations, respectively (Table S2). Considering the presence of null alleles revealed by Micro-Checker, we used FreeNA to evaluate their impact on genetic differentiation. Since null alleles had little impact on genetic differentiation for the entire dataset (*F*_ST_ including null alleles = 0.065 vs. *F*_ST_ excluding null alleles = 0.067) and for the average pairwise population differentiation (*F*_ST_ including null alleles = 0.065 vs. *F*_ST_ excluding null alleles = 0.069), the subsequent analyses were performed using the original dataset. A total of 19 out of 684 pairs of loci showed evidence of linkage disequilibrium (low = 3, mid-low = 2; mid-high = 6, high = 8). Nonetheless, only one pair (*Ph08–Ph09*) was repeated in two of the four populations (mid-high and high), indicating that most of the loci are independently inherited. Significant deviation from the Hardy–Weinberg equilibrium (HWE) was found in all populations (low = 10, mid-low = 7; mid-high = 7, high = 6), mostly due to a deficit of heterozygotes (Table S2).

### 2.3. Transferability to Phacelia brachyantha

Cross-amplification was successful for the 19 loci developed for *P. secunda*, suggesting that both species are closely related. Despite this, six out of the nineteen loci were monomorphic (*Ph14*, *Ph18*, *Ph21*, *Ph24*, *Ph25* and *Ph28*) and low allelic diversity was detected (*A* = 2.000 ± 0.265 S.E.; *A*_E_ = 1.638 ± 0.192 S.E.). However, these results may be attributed to the low sampling size (*N* = 11).

### 2.4. Within-Population Genetic Diversity Along the Altitudinal Gradient

The expected heterozygosity in the higher-elevation population (3600 m) was significantly lower (*H*_E_ = 0.434) than in the lower elevation populations (*H*_E_ = 0.524, *H*_E_ = 0.526, and *H*_E_ = 0.523 for 1600 m, 2300 m and 2800 m, respectively). Similarly, the observed heterozygosity (*H*_O_) in the lowest (1600 m) and highest elevation (3600 m) populations was significantly lower than in the mid-elevation populations (2300 m and 2800 m) (Table 1). This pattern is also reflected in the higher inbreeding coefficients detected for these populations (*F* = 0.237 at 1600 m and *F* = 0.117 at 3600 m, Table 1). Additionally, both allelic richness (*A*) and effective allelic richness (*A*_E_) were significantly lower at the highest elevation (Table 1).

### 2.5. Genetic Structure and Gene Flow Along the Altitudinal Gradient

**Figure 1:**
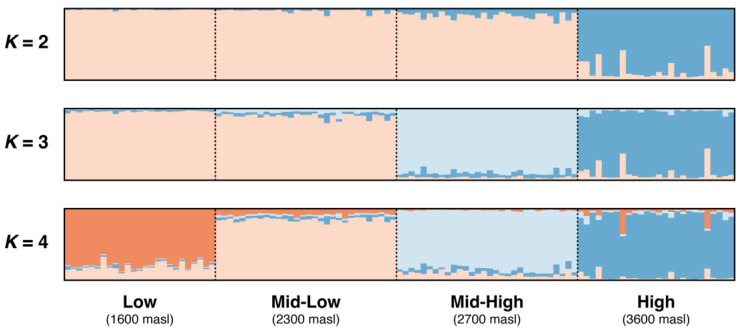
Bar plots showing the genetic structure for the 111 individuals of *Phacelia secunda* between *K* = 2 to *K* = 4, along an altitudinal gradient from the Andes of central Chile (33° S). Each vertical line in the bar plot represents an individual and is colored according to individual estimated membership coefficient (Qi) values.

The Evanno method [22] indicated an optimal *K* of two, whereas the Ln (Pr(X|K) method suggested an optimal *K* of four (Figure 1). Consequently, we analyzed the genetic structure for *K* = 2, *K* = 3 and *K* = 4. In all three clustering scenarios (Figure 1), the high-elevation population (3600 m, “high”) was consistently separated from the lower-elevation populations (1600 m “low”, 2300 m “mid-low”, 2800 m “mid-high”). Specifically, under *K* = 2, the low, mid-low and mid-high populations grouped together and separated from the high population. For *K* = 3, the low and mid-low populations formed one group, while the mid-high and high populations were separated. In contrast, *K* = 4 produced separate clusters for each elevation.

The AMOVAs performed for *K* = 2, *K* = 3 and *K* = 4 yielded similar estimates of among-population genetic differentiation (*F*_ST_ = 0.070, *F*_ST_ = 0.065, and *F*_ST_ = 0.063, respectively; Table 2). The unbiased *F*′_ST_ values for *K* = 2, *K* = 3 and *K* = 4 were also comparable and approximately doubled the uncorrected *F*_ST_ (*F*′_ST_ = 0.143, *F*′_ST_ = 0.133, and *F*′_ST_ = 0.129, respectively; Table 2), revealing substantial among-population genetic differentiation along the altitudinal gradient.

Pairwise *F*′_ST_ comparisons were consistent with the clustering proposed by STRUCTURE for *K* = 2, *K* = 3 and *K* = 4. They corroborate that the high population (3600 m) is significantly differentiated from the lower-elevation populations. For *K* = 2, the pairwise *F*′_ST_ between the low and high populations was 0.143 (Table 3 A). Under *K* = 3, the pairwise differentiation between high and mid-high (*F*′_ST_ = 0.089, Table 3B) was lower than that between the high and low + mid-low populations (*F*′_ST_ = 0.165, Table 3B). Similarly, for *K* = 4, pairwise *F*′_ST_ values between the low and mid populations (ranging from 0.053 to 0.117, Table 3C) were lower than those observed between the mid-low/mid-high groups and the high population (ranging from 0.162 to 0.196, Table 3 C).

Gene flow between low elevation sites (1600–2300 m) and the highest elevation (3600 m) were substantially lower (*Nm* = 2.538 for *K* = 3 and *Nm* = 2.050–2.539 for *K* = 4) compared to those between low elevation sites (1600–2300 m) and the mid-high population (2800 m) (*Nm* = 5.532 for *K* = 3 and *Nm* = 4.026–9.621 for *K* = 4) (Table 3C).

## 3. Discussion

The nineteen novel polymorphic microsatellites developed in this study are suitable for population genetic studies as they show little evidence of linkage, low null allele frequencies and significant polymorphism. Only one third of the markers developed for *P. secunda* were successfully amplified in the annual *P. brachyantha*, which was not expected considering the close phylogenetic relationship between the donor and target species. Their application in assessing the genetic diversity of *Phacelia secunda* along an altitudinal gradient in the Andes of central Chile confirms their utility for population genetic studies. Overall, the levels of within-population genetic diversity for the species were moderate and comparable to those reported for other Boraginaceae species, such as *Cynoglossum officinale* (*H*_O_ = 0.336 and *H*_E_ = 0.452, [26]) and *Alkanna tinctoria* (*H*_O_ = 0.512 and *H*_E_ = 0.442, [27]). Although significant population differentiation was detected (*F*_ST_ = 0.063, *p* = 0.001), these results should be interpreted with caution due to the relatively low number of populations studied here.

The SSR loci developed in this study revealed a reduction in within-population genetic diversity at higher elevations, a pattern consistent with reduced effective population size (*N*e) resulting from limited gene flow and increased genetic drift—a trend also reported in other studies, e.g., [21,22]. Similar observations by [28] indicated that genetic drift prevails at both higher and lower elevations, whereas mid-elevation populations are closer to a drift-gene flow equilibrium. Moreover, our genetic structure analysis revealed substantial differentiation among elevations, likely due to restricted gene flow. This pattern may result from genetic isolation caused by phenological segregation among elevation belts. Sequential flowering occurs along this altitudinal gradient [10,29], driven by delayed snowmelt and colder environmental conditions at higher elevations.

Interestingly, gene flow estimates support a stepping-stone mode of migration [28], in which populations that are closer in elevation exchange more migrants than those that are farther apart. In other words, these findings suggest isolation-by-distance and limited dispersal capabilities for the species along the altitudinal gradient. However, the estimates of gene flow among elevations were, in all cases, greater than one (*Nm* ranging from 2.050 to 9.621, Table 3C). This means that, on average, more than one individual (migrant) is moving from one population to another per generation, levels that are considered sufficient to prevent divergence resulting from genetic drift [30]. Considering that local adaptation typically arises from the combined effects of natural selection and restricted gene flow [15], the lack of restrictions to gene flow along the gradient implies that morphological variation in *P. secunda* might be driven primarily by phenotypic plastic responses rather than genetic divergence. Nonetheless, this conclusion could be mistaken as evidence of local adaptation to elevation despite the existence of gene flow, as has also been found [5,31]. This occurs because locally adapted genotypes can be maintained by selection even in the presence of gene flow among populations [32].

Thus, further research integrating molecular markers, reciprocal transplants and/or common garden experiments is essential to disentangle the relative contributions of genetic adaptation and plasticity to altitudinal variation in plant traits [33,34,35]. Future studies should employ higher-resolution genetic markers (e.g., SNPs via GBS, RADseq, or DArTseq) to detect signatures of selection in this and other altitudinal gradients along the Andes [18]. Additionally, other techniques such as transcriptome sequencing could also help to shed light on this question as they allow for examining genetic polymorphisms in expressed genes with a given physiological function and potential adaptative value. Epigenetic changes could also be involved in the morphological variation documented along the elevational gradient. In a recent study, Singh et al. [36] showed that *Arabidopsis thaliana* populations from different elevations are differentially methylated and that the magnitude and extent of gain and loss of DNA methylations are significantly different between low and high elevation populations. Future studies in *P. secunda* should evaluate whether epigenetic variation is related to its ability to adapt to contrasting environmental conditions along elevational gradients.

The SSR markers developed here could also be useful to conduct other investigations along altitudinal gradients but also along latitudinal gradients. For instance, they could be used to evaluate the outcrossing syndrome in *P. secunda* [7,8,9], which states that self-incompatible instead of self-compatible breeding system predominate in high-elevation ecosystems, which should be translated in high outcrossing rates. Thus, the SSRs developed here could be used to perform progeny array analyses to estimate outcrossing rates (t) [37] along altitudinal and latitudinal gradients. Furthermore, these markers may help assess the impact of the phenological pattern described by Arroyo et al. [7] on the landscape-level genetic structure of the species.

The findings of our study are highly significant in understanding how altitude influences genetic diversity and population structure in *Phacelia secunda*. The discovery that higher-elevation populations exhibit lower genetic diversity compared to those at lower elevations suggests that environmental pressures in alpine ecosystems may contribute to genetic bottlenecks or selective pressures limiting gene flow. Additionally, the observed stepping-stone migration pattern underscores the role of gradual dispersal in shaping genetic connectivity across altitudinal gradients. The newly developed polymorphic microsatellites offer a crucial resource for further investigations into the mechanisms of local adaptation, phenotypic plasticity and epigenetic modifications in mountain ecosystems. These insights are not only valuable for evolutionary biology but also for conservation strategies, as they help identify populations that may be more vulnerable to environmental changes, particularly in the face of climate change. Future studies should investigate whether the altitudinal patterns described here are repeated along the latitude.

## 4. Materials and Methods

### 4.1. Plant Materials

**Figure 2:**
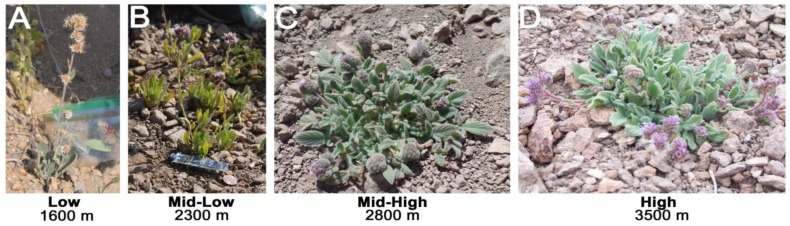
Morphological variation displayed by *Phacelia secunda* at four elevations along one altitudinal gradient in the Andes of central Chile (33° S). While the number inflorescences, the diameter of rosettes and the longitude of pubescence increases with elevation, the height of inflorescences decreases with elevation. (**A**) Low elevation (1600 m), (**B**) mid-low elevation (2300 m), (**C**) mid-high elevation (2800 m) and (**D**) high elevation (3600 m).

Plant material was collected from four elevations along an altitudinal gradient (Figure 2) near the city of Santiago, municipality of “Lo Barnechea”: low elevation (low: 1600 m) near “Yerba Loca” (33°20′33″ S, 70°20′28″ W), (ii) mid-low elevation (mid-low: 2300 m) near “Farellones” (33°21′35″ S, 70°17′43″ W), (iii) mid-high elevation (mid-high, 2800 m) near “La Parva Ski center” (33°19′43″ S, 70°17′43″ W), and (iii) high elevation (high, 3600 m) near “Cerro El Franciscano” (33°19′08″ S, 70°14′58″ W). From each population, three visually healthy young leaves per plant were collected and immediately dried in silica gel before laboratory processing. DNA from two randomly chosen individuals per elevation —a total of eight—were pooled to create the Illumina MiSeq sequencing library. A total of 111 individuals were sampled along four elevation populations: low elevation (1600 m, *n* = 25), mid-low elevation (2300 m, *n* = 30), mid-high elevation (2800 m, *n* = 30), and high elevation (3600 m, *n* = 26). From each population, a branch of one individual was taken and stored as a voucher specimen in the Herbarium of the University of Talca (low: voucher specimen No. UTAL001200, mid-low: voucher specimen No. UTAL001201, mig-high: voucher specimen No. UTAL001202, high: voucher specimen No. UTAL001203). In addition, cross-amplification was assessed in *Phacelia brachyantha* (BRA, 33°20′ S, 70°18′ W).

### 4.2. DNA Extraction and Genome Sequencing

A DNeasy^®^ Plant miniKit (Qiagen, Valencia, CA, USA) was used to extract the genomic DNA (gDNA) from dried leaves following the manufacturer’s protocol. The Illumina MiSeq sequencing was conducted by the Australomics (http://australomics.cl, accessed on 23 December 2024) sequencing service (Valdivia, Chile). The DNA extracts from all plants were quantified and standardized using a Qubit^®^ 3.0 fluorometer system (Invitrogen, Waltham, MA, USA). To detect polymorphic SSR markers, the gDNA of eight individuals was pooled to build the initial genomic library. The pool of gDNA was then normalized to 0.2 ng/μL and subsequently processed using Nextera^®^ XT DNA library preparation according to the manufacturer’s instructions. Paired-end high-throughput sequencing was performed on an Illumina HiSeq 2000 platform (Illumina, San Diego, CA, USA). Microsatellite motifs on reads longer than 80 bp were detected using the software QDD version v3.1 [38]. All sequences containing repeated motifs, with at least 95% of similitude, were compared using BLASTn [39]. This application identified a total 2015 sequences containing SSRs. The software Primer3 v4.1.0 [40] was used to design forward and reverse primers. A total of 41 putatively polymorphic SSR’s was obtained and a total of 30 primer pairs were synthesized for the subsequent screening of genetic polymorphism and genetic diversity and structure estimates. Raw reads for the successfully amplified polymorphic SSR sequences were submitted to the National Center for Biotechnology Information (NCBI), Sequence Read Archive (SRA): Accession No. PQ663010-PQ663028 (Table S1).

### 4.3. PCR Analyses and Genotyping

DNA amplification reactions were performed in a final volume of 15 μL containing: 1X PCR buffer, 1 U of Taq DNA polymerase, 1.5 mM of MgCl_2_, 0.12 mM of each dNTP and 10 μM of fluorescent forward and reverse primers. Forward primers were labeled with FAM, VIC, PET and NED fluorescent dyes from Applied Biosystems (see Table 1) to perform capillary sequencing with the ABI PRISM 310 Genetic Analyzer available at the DNA sequencing service of the Pontificia Universidad Católica de Chile (PUC), Santiago de Chile. Both alleles size and allele scoring were performed manually using the software PeakScanner v2 (Applied Biosystems, Foster City, CA, USA). SSR markers were amplified using the following protocol: an initial denaturation step at 95 °C for 5 min, 35 cycles consisting of a denaturation step at 95 °C for 30 s, specific annealing temperature (Ta) of each primer pair for 30 s (Table 1), an extension step at 72 °C for 35 s, followed by a final step of DNA extension at 72 °C for 7 min. Each PCR reaction was performed separately and then assigned to one of five mixes before capillary sequencing (Table S1).

### 4.4. Microsatellite Loci Characterization

The software GENALEXv6.51b2 [41] was used to calculate the following population genetic statistics: total number of alleles (*A*_T_), number of alleles per population (*A*), number effective alleles per population (*A*_E_), observed heterozygosity (*H*_O_), expected heterozygosity (*H*_E_), inbreeding coefficient (*F*), and Hardy–Weinberg equilibrium (HWE) significance. Linkage disequilibrium (LD) was evaluated using GENEPOP v4.2 [42]. The frequencies of null alleles were calculated via Micro-Checker v2.2.3 [43] using Brookfield’s estimator 1 [44]. The significance of null allele frequencies was assessed using Bonferroni correction to avoid the effect of multiple comparisons. FreeNA [45] was used to evaluate the impact of null alleles on among-populations differentiation.

### 4.5. Transferability Assessment

The transferability of the newly SSR loci developed for *P. secunda* to its annual congener *P. brachyantha* was assessed by performing cross amplifications on eleven individuals collected near the city of Santiago.

### 4.6. Within-Population Genetic Diversity Along the Altitudinal Gradient

To apply the novel SSR markers developed here, we studied the within-population and among-population genetic diversity along an altitudinal gradient in the Andes of central Chile (33° S). To investigate the changes in within-population genetic diversity estimators (*A*, *A*_E_, *H*_O_, *H*_E_ and *F*) along the gradient, we implemented the W-test (Wilcoxon sign-rank test, *p* < 0.05) as an a posteriori multiple-comparison analysis to estimate significant differences among populations.

### 4.7. Genetic Structure and Gene Flow Along the Altitudinal Gradient

The number of genetic groups (*K*) was evaluated in STRUCTURE v2.3.4 [46,47,48]. A total of 600,000 replicates were analyzed by performing an MCMC analysis considering a burn-in period of 300,000 parameters, and the results were obtained based on 15 runs (K = from 1 to 4). The structuring parameters were calculated for each K value under the admixture model and using locality of origin information as LocPrior [46,47]. The most likely number of populations inferred by STRUCTURE (*K*S; where S denotes STRUCTURE) was evaluated considering the value of the log-likelihood of the observed data (LnP [D]) and the second-order change rate of the log-likelihood of the data in different runs of K (ΔK) described in [49] using the online platform of STRUCTURE HARVESTER v0.6.94 [50]. Results were summarized and compared in CLUMPAK v1.1 [51]. The results of the STRUCTURE analysis were visualized using bar plots, where each individual is represented by a vertical bar divided into *K* colored segments. Each segment corresponds to a Q value (Qi), which represents the estimated proportion of the individual’s genome assigned to the *i*-th genetic cluster. These values provide insight into the individual’s genetic ancestry and the degree of admixture among clusters, with all Qi values summing to 1 for each individual.

The genetic structure was also examined through hierarchical analyses of molecular variance (AMOVAs) using GENALEXv6.51b2 [41]. From the AMOVAs, *F*_ST_ [30,52,53] was calculated for the optimal *K* suggested by STRUCTURE. To cope with the bias due to the low number of populations studied here, we calculated the unbiased *F*′_ST_ developed by [54]. This statistic is also called *G*″_ST_, as it is based on G_ST_ calculation. In practice, *F*′_ST_ is calculated by dividing *F*_ST_ by the maximum possible *F_S_*_T_ (*F*_ST_ max), which is (1 − c*H*_S_), where c*H*_S_ is the corrected average expected heterozygosity across loci (*H*_S_) adjusted for a low sampling size according to [55]. This estimator of population differentiation is unbiased for low sampling size and low number of populations (*K*), and in addition, it also corrects the dependency of *F*_ST_ on expected heterozygosity *H*_S_, a bias that increases with the amount of within-population diversity (*H*_S_) and is greater in multiallelic markers such as microsatellites, in which the maximum possible value of *F*_ST_ is not necessarily equal to one [54]. As stated by [54], this statistic should be calculated whenever the number of sampled populations is small, especially for pairwise comparisons (see [54], page 10). The significance of these estimators was estimated based upon 1000 permutations. Pairwise *F*′_ST_ were used to analyze the differences between localities from different elevations along the altitudinal gradient. Finally, gene flow (*Nm*) was calculated from *F_S_*_T_ using the formula *Nm* = (1 − *F*′_ST_)/(4 *F*_ST_) according to [54]. This calculation assumes an equilibrium Island model and provides an estimate of the gene flow that is unaffected by *H*_S_ as it combines *F*_ST_ and *F*′_ST_.

## 5. Conclusions

The nineteen novel SSR markers developed here for the widely distributed *Phacelia secunda* fulfill the requirements for their use in population genetic studies. Their application along a 2000 m altitudinal gradient revealed a reduction in within-population genetic diversity at the highest elevation and significant differentiation among elevations, consistent with a stepping-stone model of gene flow. These findings indicate that local adaptation along the gradient is possible; however, further research incorporating reciprocal transplants or common garden experiments is necessary to confirm this. Moreover, the new SSR loci provide a valuable tool for a wide range of studies, including investigations of breeding systems, landscape genetics, and fine-scale population structure.

## Figures and Tables

**Table 1:** Summary of the estimated genetic diversity for *Phacelia secunda*, using the newly developed 19 SSR loci, along an altitudinal gradient in the Andes of central Chile (33° S). Different bold lowercase letters indicate significant differences between populations (Wilcoxon test, *p* < 0.05).

Population	Elevation (m)		*A*	*A*_E_	*H*_O_	*H*_E_	*F*
Low	1600	Mean	4.985 **^a^**	2.810 **^a^**	0.394 **^a^**	0.524 **^a^**	0.237 **^a^**
		SE	0.529	0.388	0.045	0.055	0.067
Mid-Low	2300	Mean	4.947 **^a^**	2.587 **^a^**	0.504 **^b^**	0.526 **^a^**	0.025 **^b^**
		SE	0.543	0.294	0.049	0.050	0.052
Mid-High	2800	Mean	4.842 **^a^**	2.847 **^ab^**	0.472 **^a^**	0.523 **^a^**	0.062 **^b^**
		SE	0.563	0.407	0.051	0.054	0.065
High	3500	Mean	4.263 **^b^**	2.053 **^b^**	0.387 **^a^**	0.434 **^b^**	0.117 **^ab^**
		SE	0.396	0.186	0.055	0.051	0.064
Total	All	Mean	**4.737**	**2.574**	**0.439**	**0.502**	**0.110**
		SE	0.253	0.166	0.026	0.026	0.032

*A* = number of alleles, *A*_E_ = number of effective alleles, *H*_O_ = observed heterozygosity, *H*_E_ = expected heterozygosity, *F* = inbreeding coefficient.

**Table 2:** Summary of the AMOVA results for *K* = 2 to *K* = 4 in *Phacelia secunda* along the altitudinal gradient from the Andes of central Chile (33° S).

Source of Variation	d.f.	Sum of Squares	Mean Square	Estimated Variance	Percentage of Variation
**(A) *K* = 2—Group 1: Low + Mid-Low + Mid-High; Group 2: High**					
Among populations	1	34.880	34.880	0.375	7%
Within populations	220	1095.692	4.980	4.980	93%
Total	221	1130.572		5.356	100%
*F*_ST_ = 0.070, *p* = 0.001; *F*_ST_ max = 0.490; *F*′_ST_ = 0.143, *p* = 0.001					
**(B) *K* = 3—Group 1: Low + Mid-Low; Group 2: Mid-High; Group 3: High**					
Among populations	2	56.986	28.493	0.339	6%
Within populations	219	1073.586	4.902	4.902	94%
Total	221	1130.572		5.241	100%
*F*_ST_ = 0.065, *p* = 0.001; *F*_ST_ max = 0.487; *F*′_ST_ = 0.133, *p* = 0.001					
**(C) *K* = 4—Each sampling site is considered a population**					
Among populations	3	69.062	23.021	0.328	6%
Within populations	218	1061.511	4.869	4.869	94%
Total	221	1130.572		5.197	100%
*F*_ST_ = 0.063, *p* = 0.001; *F*_ST_ max = 0.488; *F*′_ST_ = 0.129, *p* = 0.001					

**Table 3:** Pairwise population differentiation (*F*′_ST_, below diagonal) and gene flow (*Nm*, above diagonal) for *K* = 2 to *K* = 4 in *Phacelia secunda* along the altitudinal gradient from the Andes of central Chile (33° S).

**(A) *K* = 2**				
**Population**		**Low + Mid-Low + Mid-High** **(1600 + 2300 + 2800 m)**		**High** **(3600 m)**
Low + Mid-Low + Mid-High (1600 + 2300 + 2800 m)		–		4.767
High (3600 m)		0.143		–
**(B) *K* = 3**				
**Population**		**Low + Mid-Low** **(1600 + 2300 m)**	**Mid-High** **(2800 m)**	**High** **(3600 m)**
Low + Mid-Low (1600 + 2300 m)		–	5.552	2.538
Mid-High (2800 m)		0.089	–	2.539
High (3600 m)		0.165	0.162	–
**(C) *K* = 4**				
**Population**	**Low** **(1600 m)**	**Mid-Low** **(2300 m)**	**Mid-High** **(2800 m)**	**High** **(3600 m)**
Low (1600 m)	–	9.621	4.026	2.050
Mid-Low (2300 m)	0.053	–	5.586	2.409
Mid-High (2800 m)	0.117	0.088	–	2.539
High (3600 m)	0.192	0.196	0.162	–

## Data Availability

All data generated during this study are included in this published article, and the raw data used or analyzed during the current study are available from the NCBI (https://submit.ncbi.nlm.nih.gov/, accessed on 21 February 2025).

## Supplementary Materials

The following supporting information can be downloaded at: https://www.mdpi.com/article/10.3390/plants14071135/s1, Table S1: Characteristics of the 19 microsatellite markers developed for *Phacelia secunda.*; Table S2: Results of the initial primer screening of 19 polymorphic loci in four populations of *Phacelia secunda.*

## Abbreviations

The following abbreviations are used in this manuscript:

HTS	High-throughput sequencing
SSRs	Simple sequence repeats
SNPs	Single nucleotide polymorphisms
HWE	Hardy–Weinberg equilibrium
LD	Linkage disequilibrium
