# Supplementary material for: Development and Application of Novel SSR Markers to Assess the Genetic Diversity and Population Structure of Phacelia secunda Along an Altitudinal Gradient in the Central Chile Andes
Source: Plants (Basel). 2025 Apr 5;14(7):1135. doi: 10.3390/plants14071135 (PMC11991144; doi:10.3390/plants14071135)
Supplement: Supplementary file 1 [file plants-14-01135-s001.zip › Supplementary Tabla S1.pdf]

Supplementary Table S1. Characteristics of the 19 microsatellite markers developed for *Phacelia secunda.*

Locus	Primer sequence (5′-3′)	Repeated motif	Allele size (bp)	Fluorescent Dye	Ta (ºC)	Mix *	GenBank accession no.
Ph01	F: GTGCGTTTGAGGAAGGACTC	$(AGG)_5$	255	NED	56	4	PQ663010
	R: ATCGCCACCGGTAGTTGTAG						
Ph02	F: CCTGTTAGTGTAACTTGGAGCG	$(AAG)_5$	168	NED	51	1	PQ663011
	R: CCCAAGCAGAATGTTAGTAATCC						
Ph03	F: CTGCCCTCGGAAAGTTAGTG	$(AG)_7$	272	6-FAM	56	4	PQ663012
	R: TTCAGGCAGCAGATGCAGTA						
Ph04	F: GCCCATGTCATTGATTCCTT	$(AG)_9$	260	6-FAM	53	2	PQ663013
	R: GCTGGTGAAGAAAGAGGTGG						
Ph05	F: CCGCAGGAATTTCATGTCTT	$(AG)_8$	254	6-FAM	54	5	PQ663014
	R: GAGTGTGAGACACCAAGTGGAA						
Ph07	F: CCGCTCTAACTAACCCTTGC	$(AG)_9$	155	NED	50	3	PQ663015
	R: CAAATGCTGAGGCTTGGATT						
Ph08	F: CACGAGACATCACCGAGTTAAA	$(AAG)_6$	238	PET	57	4	PQ663016
	R: GCTCTGCTATGTTCTGTCTCCTC						
Ph09	F: CTGCGTGGGTGACATACAAC	$(AAG)_6$	226	NED	52	2	PQ663017
	R: CCCAAGTCTGCTGATGATGA						
Ph14	F: ATTGATCCACCACCACGAAT	$(ATC)_6$	129	VIC	53	2	PQ663018
	R: CACAGATAGAGCATGGAGCTG						
Ph15	F: ACCGTAGGAGCCATGTGGT	$(AG)_5$	278	VIC	52	5	PQ663019
	R: GACCCATTTGAGATGTTGGTG						

Ph17	F: GCAAACACCTCAACTAGCCC<br>R: TGCCAATAGAAAGGAGGGAG	$(AT)_5$	127	NED	51	4	PQ663020
Ph18	F: GCATCATAGCCGCAATTGTA<br>R: ATCCCAAATCCTTCACACCC	$(ATC)_6$	171	6-FAM	50	2	PQ663021
Ph20	F: GGACAAGTTGCACATCGAGTT<br>R: TGCATAACCCTGCATACCCT	$(AG)_7$	249	PET	51	3	PQ663022
Ph21	F: TGACCAATGATTTCCAAACAC<br>R: TGACATTTCTGTTCCTGCTTTG	$(ATC)_6$	217	NED	52	5	PQ663023
Ph22	F: TGGAAGTCATCCGTCTGAAG<br>R: CATATTTCGGCCTCCCATATC	$(AGG)_6$	317	VIC	56	1	PQ663024
Ph24	F: TTCTAAGCATGCCATCAACG<br>R: TACTACACAACACAACACAACAAGTG	$(AC)_6$	90	VIC	51	4	PQ663025
Ph25	F: CCGTTGTCTCACACTCTTCATC<br>R: GCTTCTCTTTGTAGGTAACGAAA	$(AG)_6$	108	VIC	51	3	PQ663026
Ph27	F: GTCAGATTCCTTCAGCAGCC<br>R: TGGATGCTGATCGCCATTAT	$(ACC)_5$	130	VIC	54	1	PQ663027
Ph28	F: CCAAACAACCTAAACCCTCAGA<br>R: GTTTGGGTAAGCGAGTGTCC	$(ATC)_5$	290	6-FAM	56	3	PQ663028

* = PCR reactions were made independently but were sequenced in five different mixes.
