# Supplementary material for: Development and Application of Novel SSR Markers to Assess the Genetic Diversity and Population Structure of Phacelia secunda Along an Altitudinal Gradient in the Central Chile Andes
Source: Plants (Basel). 2025 Apr 5;14(7):1135. doi: 10.3390/plants14071135 (PMC11991144; doi:10.3390/plants14071135)
Supplement: Supplementary file 1 [file plants-14-01135-s001.zip › Supplementary Table S2.pdf]

Supplementary Table S2. Results of the initial primer screening of 19 polymorphic loci in four populations of *Phacelia secunda.*

	**Low – 1600 m (N = 25)**							**Mid-Low – 2300m (N = 30)**					
Locus	A_T	A	A_E	H_O	H_E	F	*Null*	A	A_E	H_O	H_E	F	*Null*
Ph01	7	4	2.023	0.320	0.506	0.367	0.170	6	3.214	0.633	0.689	0.081	
Ph02	6	3	1.084	0.080	0.078	-0.031**		5	1.146	0.133	0.128	-0.043	
Ph03	8	6	5.061	0.720	0.802	0.103***		5	3.550	0.633	0.718	0.118	
Ph04	7	6	3.754	0.360	0.734	0.509**	0.242	6	3.789	0.567	0.736	0.230***	0.115
Ph05	12	10	6.281	0.600	0.841	0.286**	0.137	9	3.607	0.467	0.723	0.354***	0.179
Ph07	11	8	5.787	0.560	0.827	0.323***	0.159	8	3.689	0.433	0.729	0.405**	0.193
Ph08	12	10	5.787	0.360	0.827	0.565	0.274	11	6.383	0.900	0.843	-0.067***	
Ph09	6	3	1.378	0.320	0.274	-0.166		4	1.363	0.267	0.266	-0.002	
Ph14	4	4	1.997	0.320	0.499	0.359*		3	2.341	0.433	0.573	0.243	
Ph15	5	5	2.220	0.480	0.550	0.127	0.148	4	1.846	0.533	0.458	-0.164	
Ph17	4	3	1.224	0.200	0.183	-0.092		3	1.144	0.133	0.126	-0.057	
Ph18	5	5	2.148	0.680	0.534	-0.272		4	2.403	0.833	0.584	-0.427*	
Ph20	3	2	1.083	0.000	0.077	1.000***	0.179	3	1.665	0.333	0.399	0.166	
Ph21	3	3	1.705	0.360	0.414	0.130		2	1.724	0.533	0.420	-0.270	
Ph22	6	4	2.341	0.280	0.573	0.511***	0.235	6	2.808	0.700	0.644	-0.087**	
Ph24	5	3	1.788	0.240	0.441	0.456	0.206	3	1.665	0.267	0.399	0.332	
Ph25	6	4	2.822	0.640	0.646	0.009***		5	2.723	0.800	0.633	-0.264***	
Ph27	6	6	2.900	0.520	0.655	0.206**		5	2.659	0.667	0.624	-0.069	
Ph28	6	4	2.006	0.440	0.502	0.123***		2	1.427	0.300	0.299	-0.002	
**Mean**	**5.421**	**4.985**	**2.810**	**0.394**	**0.524**	**0.237**		**4.947**	**2.587**	**0.504**	**0.526**	**-0.025**	
**(S.E.)**	(0.630)	(0.529)	(0.388)	(0.045)	(0.055)	(0.069)		(0.543)	(0.294)	(0.052)	(0.049)	(0.042)	

Asterisks indicate significant differences from HWE equilibrium (based in Chi2 tests) * = P < 0.05, ** = P < 0.01; *** = P < 0.001, NS = Not significant. Population abbreviations: **Low** = 1600 m, **Mid-Low** = 2300 m, **Mid-High** = 2800, **High** = 3600 m. N = Sample size, A_T = total number of alleles, A = number of alleles, A_E = number effective alleles, H_O = observed heterozygosity, H_E = expected heterozygosity, F = inbreeding coefficient, *Null* = null alleles frequency calculated using Brookfield's estimator 1, according to [18].

Table S2 (Continued)

	**Mid-High (*N* = 30)**						**High – 3600 m (*N* = 26)**					
Locus	*A*	*A*E	*H*O	*H*E	*F*	*Null*	*A*	*A*E	*H*O	*H*E	*F*	*Null*
Ph01	5	1,711	0.400	0.416	0.037		5	2.946	0.538	0.661	0.185	
Ph02	3	1,106	0.100	0.096	-0.040		2	1.122	0.115	0.109	-0.061	
Ph03	7	5,625	0.667	0.822	0.189***	0.085	4	1.926	0.269	0.481	0.440**	0.189
Ph04	7	5,000	0.700	0.800	0.125		5	2.018	0.308	0.504	0.390	0.176
Ph05	10	5,233	0.733	0.809	0.093*		7	2.817	0.500	0.645	0.225	
Ph07	9	6,272	0.567	0.841	0.326***	0.159	7	3.953	0.731	0.747	0.022	
Ph08	9	5,902	0.467	0.831	0.438***	0.213	8	2.971	0.654	0.663	0.014**	
Ph09	3	1,407	0.267	0.289	0.079		3	1.124	0.038	0.110	0.651***	0.157
Ph14	4	2,025	0.200	0.506	0.605***	0.254	4	1.437	0.115	0.304	0.620***	
Ph15	3	2,270	0.633	0.559	-0.132*		3	1.671	0.462	0.402	-0.149	0.220
Ph17	3	1,265	0.200	0.209	0.045		2	1.039	0.038	0.038	-0.020	
Ph18	3	2,052	0.933	0.513	-0.820***		4	2.288	0.846	0.563	-0.503*	
Ph20	3	2,965	0.500	0.663	0.246		3	2.143	0.385	0.533	0.279	
Ph21	3	1,268	0.200	0.212	0.055		3	2.364	0.500	0.577	0.133	
Ph22	3	2,560	0.600	0.609	0.015		5	2.376	0.577	0.579	0.004	
Ph24	5	1,656	0.367	0.396	0.074		4	1.219	0.192	0.180	-0.070	
Ph25	2	1,991	0.467	0.498	0.063		3	1.465	0.308	0.317	0.030	
Ph27	4	2,359	0.633	0.576	-0.099		6	2.914	0.577	0.657	0.122*	
Ph28	6	1,425	0.333	0.298	-0.117		3	1.215	0.192	0.177	-0.088	
**Mean**	**4.842**	**2.847**	**0.472**	**0.523**	**0.062**		**4.263**	**2.053**	**0.387**	**0.434**	**0.117**	
**(S.E.)**	(0.563)	(0.407)	(0.051)	(0.055)	(0.065)		(0.396)	(0.186)	(0.055)	(0.051)	(0.064)	
